# Theta tACS impairs episodic memory more than tDCS

**DOI:** 10.1038/s41598-022-27190-y

**Published:** 2023-01-13

**Authors:** Nicholas W. G. Murray, Petra L. Graham, Paul F. Sowman, Greg Savage

**Affiliations:** 1 School of Psychological Sciences, Macquarie University, Australian Hearing Hub, Level 3, Sydney, NSW 2109 Australia; 2 School of Mathematical and Physical Sciences, Macquarie University, Sydney, Australia

**Keywords:** Cognitive neuroscience, Learning and memory

## Abstract

Episodic memory deficits are a common consequence of aging and are associated with a number of neurodegenerative disorders (e.g., Alzheimer’s disease). Given the importance of episodic memory, a great deal of research has investigated how we can improve memory performance. Transcranial electrical stimulation (TES) represents a promising tool for memory enhancement but the optimal stimulation parameters that reliably boost memory are yet to be determined. In our double-blind, randomised, sham-controlled study, 42 healthy adults (36 females; 23.3 ± 7.7 years of age) received anodal transcranial direct current stimulation (tDCS), theta transcranial alternating current stimulation (tACS) and sham stimulation during a list-learning task, over three separate sessions. Stimulation was applied over the left temporal lobe, as encoding and recall of information is typically associated with mesial temporal lobe structures (e.g., the hippocampus and entorhinal cortex). We measured word recall within each stimulation session, as well as the average number of intrusion and repetition errors. In terms of word recall, participants recalled fewer words during tDCS and tACS, compared to sham stimulation, and significantly fewer words recalled during tACS compared with tDCS. Significantly more memory errors were also made during tACS compared with sham stimulation. Overall, our findings suggest that TES has a deleterious effect on memory processes when applied to the left temporal lobe.

## Introduction

Our ability to recall context-based information, such as events and personal experiences, is fundamental to everyday functioning^1,2^. This type of memory, known as episodic memory, facilitates learning by allowing us to draw upon previous experiences and apply them to new situations or tasks. Impaired episodic memory, a common consequence of aging and neurodegenerative disorders (e.g., Alzheimer’s disease [AD])^3,4^, can be extremely debilitating. As the formation and consolidation of memories is structurally associated with the temporal lobe^5,6^, memory deficits are also common in certain focal epilepsies, where seizure activity involves temporal regions and/or associated networks (i.e., temporal lobe epilepsy [TLE])^7,8^. Impaired episodic memory in these disorders has been associated with a shared physiological process, resulting from selective neuronal death in memory circuitry over time (for a review see^9^). This selective atrophy has been associated with desynchronisation of functional network activity^10^. Accordingly, research has increasingly focussed on modulating functional network activity when attempting to improve cognition, often using transcranial electrical stimulation (TES).

The therapeutic use of electrical stimulation is an area that has received a great deal of research in recent years, particularly in the context of memory. A large portion of the research exploring stimulation and cognition has used transcranial direct current stimulation (tDCS), where a low electrical current (typically 1–2 mA) is introduced to the scalp via two electrodes^11^. Overall, this research has garnered mixed results, with findings ranging from improved recall^12,13^ to null or even deleterious effects on memory due to stimulation^14,15^. A recent systematic review and meta-analysis from Galli et al.^16^ details these mixed findings further in the context of episodic memory. While these findings may cast doubt upon the utility of tDCS when attempting to improve memory performance, it does demonstrate the impact that TES can have on cognition. One explanation for the mixed findings is that tDCS does not allow for stimulation at specific frequencies known to correspond with successful performance of cognitive tasks.

When performing any cognitive task, synchronised neural oscillations facilitate the local and distal communication of brain regions involved in the functional network^17^. During memory processes, there is a synchronised coupling of theta (4–12 Hz) and gamma activity (low frequency gamma, 25–45 Hz; high frequency gamma, 50–120 Hz), known as phase amplitude coupling (PAC), which primarily occurs between the mesial temporal lobe (MTL) and the prefrontal cortex (PFC)^18,19^. While both regions appear to be involved in effective recall of information, this cognitive process has primarily been localised to the MTL, where theta activity is proposed to facilitate communication with other distal structures, such as the PFC^20^. Reduced coherence between theta and gamma activity has also been linked to poor memory performance in TLE and AD^20,21^. While some research has demonstrated that tDCS can increase PAC^22,23^, tDCS is, by definition, not frequency selective (i.e., theta or gamma). This has ultimately led researchers to explore other stimulation modalities that allow specific frequencies (i.e., those associated with successful memory performance) to be applied to certain brain regions, such as transcranial alternating current stimulation (tACS).

**Figure 1:**
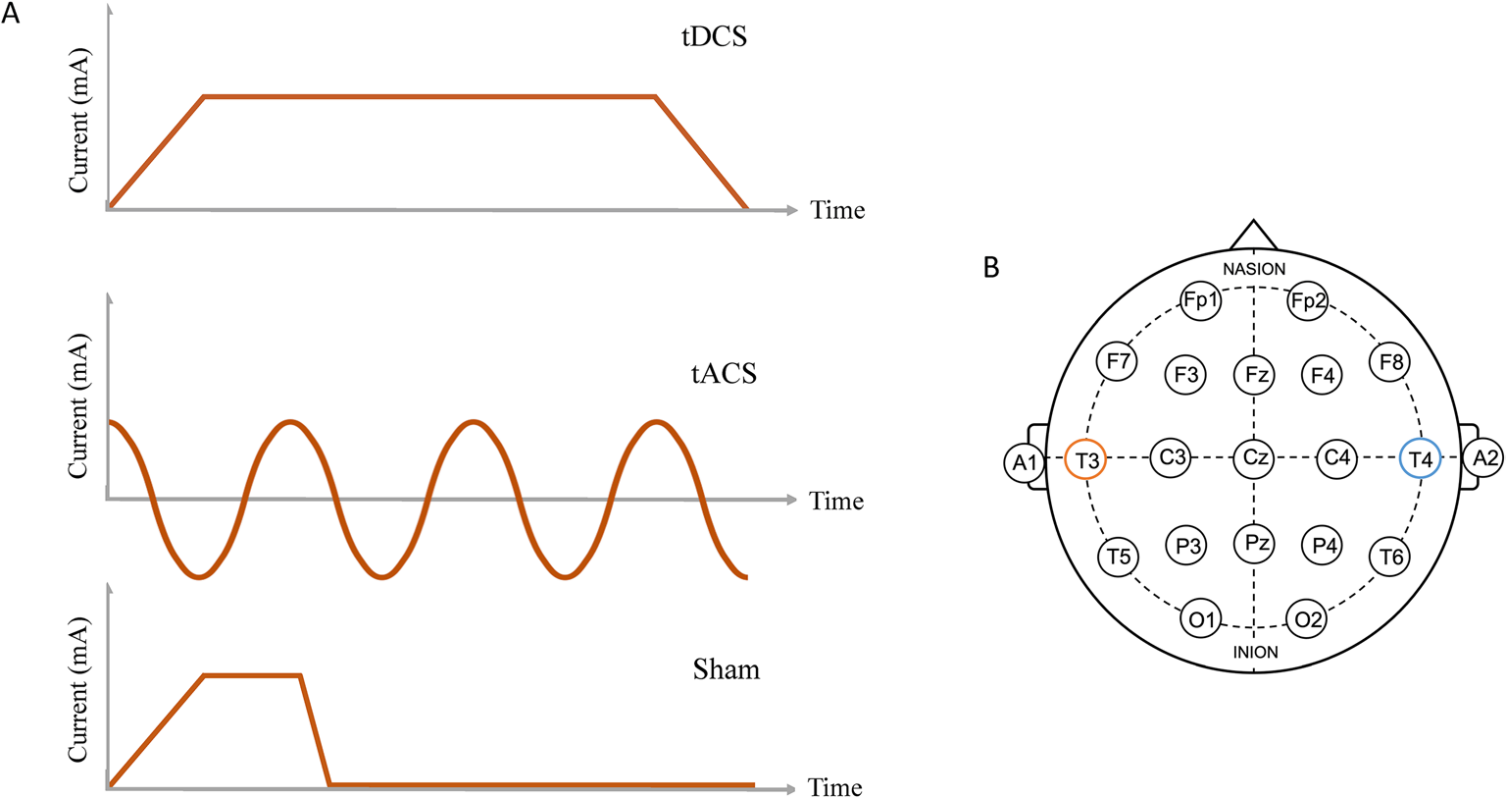
(**A**) Different stimulation types utilised within the study: transcranial direct current stimulation (tDCS), transcranial alternating current stimulation (tACS) and sham stimulation. (**B**) International 10–20 EEG electrode arrangement with the anodal electrode (T3) in orange and the cathodal/reference electrode (T4) in blue.

The mechanism by which tDCS and tACS apply electrical current to the brain is fundamentally different. tDCS applies electrical currents through multiple scalp electrodes and is thought to modulate the excitability of neuronal populations, depending on whether a positive charge (anodal, excitatory) or negative charge (cathodal, inhibitory) is applied. While tACS also applies electrical current through scalp electrodes, it utilises an oscillating sinusoidal current (i.e., more closely resembling normal brain activity) to modulate rhythmic cortical network activity^10^. The difference between these stimulation types is illustrated in Fig. 1. A few studies have investigated the comparative effects of tDCS and tACS in cognition, some of which have found greater improvement in cognition following tACS. Röhner et al.^24^ compared anodal tDCS and theta tACS applied over the dorsolateral PFC (DLPFC) during a working memory task. Their findings suggested that tACS resulted in reduced reaction time for correct responses compared to tDCS. Reinhart and Nguyen^10^ also investigated working memory performance when applying theta tACS over the left DLPFC and left temporal cortex concurrently and found that not only did tACS improve working memory performance, but it also increased the coherence of theta-gamma activity between these brain regions. Lang et al.^2^ compared the effects of tDCS and theta tACS using a visual associative memory task. Their findings showed that tACS improved associative memory performance above and beyond that of tDCS (greater recall and correct rejection of items). In contrast to Röhner et al.^24^, they applied stimulation over the right fusiform gyrus rather than the DLPFC, suggesting that this region is involved in the encoding and recall of visual information.

In the present study, we aimed to investigate whether anodal tDCS and theta tACS (using 6 Hz stimulation) can improve episodic verbal memory performance in healthy adults, when applied to the left temporal lobe. We hypothesised that 1) both tDCS and theta tACS would improve memory performance compared to sham, as the literature suggests that the introduction of electrical activity to the temporal lobe is typically associated with improved memory performance, and 2) that tACS would result in a greater improvement in recall performance compared to tDCS, through the use of theta frequency stimulation. An understanding of the comparative effects of these stimulation methods on memory performance is vital when investigating the therapeutic effects of stimulation in clinical cohorts, such as AD and epilepsy.

## Method

### Design

This was a double-blind, within groups, randomised, sham-controlled study comparing the difference between anodal tDCS, theta tACS (i.e., 6 Hz tACS) and sham stimulation on a verbal memory task.

### Participants

Forty-two healthy participants aged 18–50 (age mean ± SD = 23.3 ± 7.7 years; education mean ± SD = 14.12 ± 2.6 years; 36 females; 2 left-handed [both female]) were recruited from Macquarie University using an online participant pool. Each participant provided written informed consent to take part in the study and was remunerated with course credits. Participants were included providing they were over the age of 18, had no history of neurological or psychiatric illness and learned English as a first language. All procedures were approved by the Macquarie University Human Research Ethics Committee and conducted according to the National Statement on Ethical Conduct in Human Research (2007). All methods were performed in accordance with the relevant guidelines and regulations.

### Verbal memory task

Episodic memory was assessed using a delayed free recall task in which participants were instructed to study and recall a series of lists of 12 words. A pool of 30 word lists was compiled, each comprising 15 high frequency English nouns (sourced from http://memory.psych.upenn.edu/WordPools); each study list was balanced in terms of concreteness and familiarity of words. For each participant, a test set of 24 lists was randomly selected and allocated evenly across the three blocked stimulation conditions (i.e., eight lists per session), and within each list 12 words were randomly selected to form a test list. Thus, each participant learned a unique randomisation of 96 words per session.

Words were presented on screen for 1600 ms followed by a 2000 ms blank interval between each word. Following the final word of the list, participants were instructed to complete a page of simple arithmetic equations for 20 s, to prevent the rehearsal of list items. Following this task, participants were given 45 s to recall as many of the 12 target words as possible, in any order. We specifically measured short-term recall following the distractor task, as previous research has demonstrated a robust association between retroactive interference and MTL dysfunction^25,26^. The total number of errors for each trial was also noted and included any intrusion errors (i.e., unrelated, or semantically related words that were not on the list) or repetitions (i.e., words repeated on the same list or words from a previous list). To avoid any carryover effects, each session was separated by at least 72 h, with most sessions separated by a week, in accordance with the general recommendations for non-invasive stimulation studies^27^.

### Stimulation

TES was delivered using a neuroConn GmbH DC-Stimulator Plus (neuroConn GmbH, Ilmenau, Germany), which allows for programmable direct and alternating stimulation. Two electrodes enclosed in saline-soaked sponges were placed over the left (T3 [5 × 5 cm]) and right (T4 [6 × 8.5 cm]) temporal lobes, based on the 10–20 EEG system. The anode was place over T3, while the cathodal/reference electrode was over T4. This montage has previously been used to target mesial temporal regions in other studies^28–30^. Stimulation parameters for each condition were as follows: tACS—6 Hz, 7200 cycles, 0° phase, 1.5 mA amplitude and 10 s fade in/out; tDCS—1.5 mA, 10 s fade in/out; sham—1.5 mA direct current (i.e., tDCS) applied for 30 s following 10 s fade in/out. Stimulation lasted approximately 20 min in each session and adhered to established safety protocols^11,31^. Double blinding was achieved by randomly allocating each stimulation condition to a pre-set function (i.e., A, B or C) and using a set of unidentifiable codes that correspond to either stimulation or sham stimulation. The pre-set functions were programmed by an impartial second party who also designated active and sham stimulation codes depending on the condition. Average impedance was calculated for each session by taking three measures of impedance during stimulation and was kept below 10 kΩ for all sessions^32^.

### Modelling of stimulation

**Figure 2:**
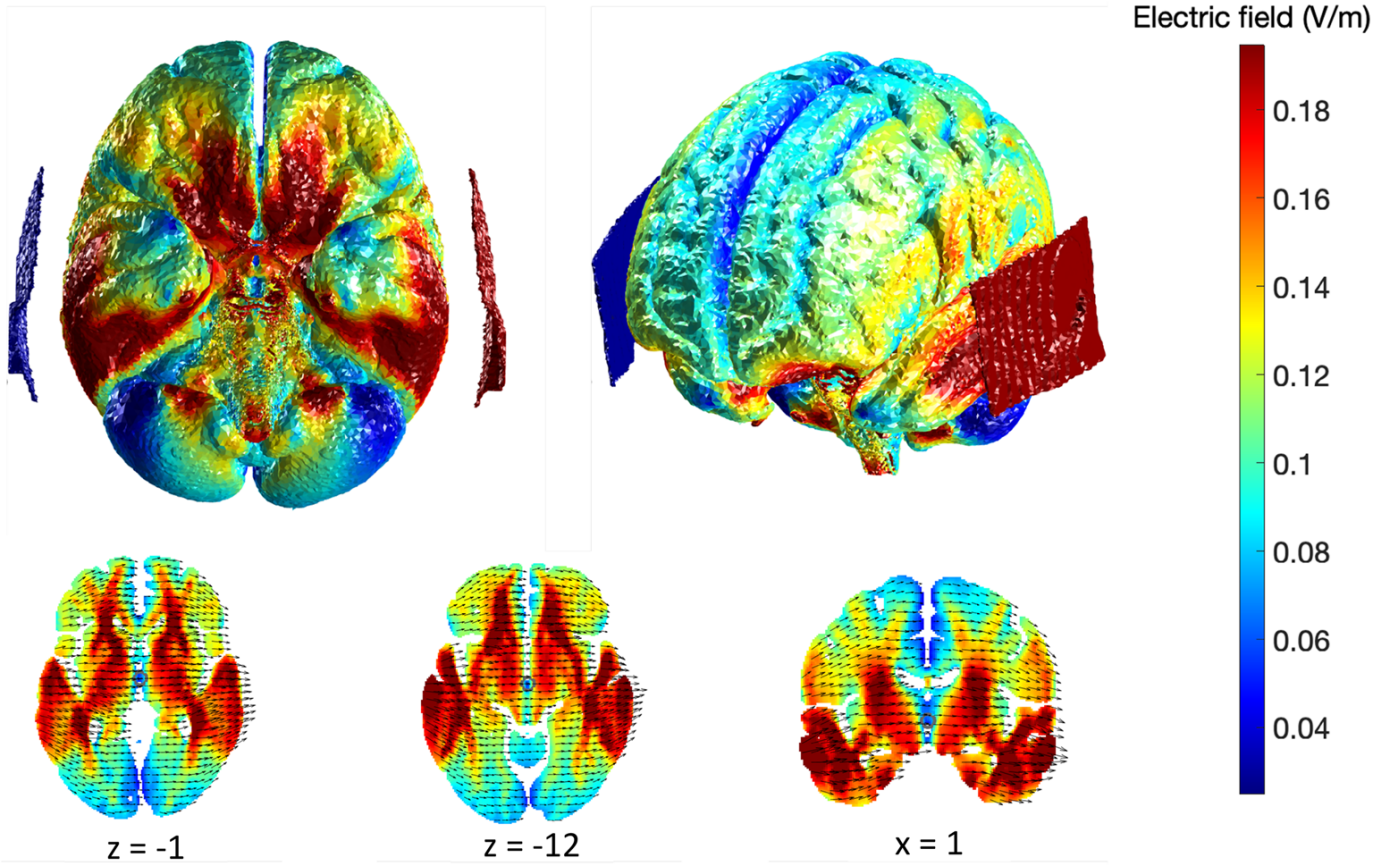
Modelling of predicted electrical fields based on tDCS stimulation parameters. Red indicates regions of maximal current density. Red electrode pad represents the anodal electrode (T3), blue represents the cathodal/reference electrode (T4).

We used computational modelling to depict the predicted effect of stimulation on the brain, in the absence of individual MRI scans. The New York Head model was utilised within ROAST (Realistic, vOlumetric Approach to Simulate Transcranial electric stimulation) to predict the electrical fields induced by stimulation, based on our stimulation parameters (i.e., electrode size and placement, current intensity)^33,34^. Predictive modelling of electrical fields has been utilised in a number of studies where individual MRIs are not available^35,36^. The model uses a high-resolution MRI which is segmented for six tissue types (scalp, skull, cerebrospinal fluid, grey matter, white matter, and air cavities) at 0.5 mm resolution^37^. The models presented in Fig. 2 indicate that our stimulation parameters likely engaged our region of interest (i.e., mesial temporal lobe structures).

### Additional questionnaires

At the end of each session participants were asked to indicate the perceived intensity of stimulation (1–10; very low-very intense). They also completed a side-effects questionnaire which required participants to report the presence and intensity of a list of typical side-effects (e.g., itchiness, burning, iron taste, etc.). This questionnaire also asked participants to estimate when stimulation began, how long it lasted and whether they felt it impacted their performance on the word recall task. These questions were used to determine the effectiveness of sham stimulation.

### Statistical analyses

All statistical analyses were performed using STATA 17^38^. A repeated measures analysis of variance (ANOVA) was used to determine the impact of stimulation type on memory performance, with stimulation type as a within-subjects factor (i.e., sham, tDCS and tACS). Using this design, an a-priori sample size calculation with power equal to 80%, a significance level of 5% and a moderate effect size (*f* = 0.25), indicated a minimum sample size of 30 participants. Forty-two participants were recruited for the following study, as noted in Section “Participants”. This analysis was also used when analysing the impact of stimulation on the number of intrusion and repetition errors, as well as certain stimulation factors (i.e., average impedance and perceived intensity of stimulation). Post hoc tests of significant pairwise effects were conducted using Tukey’s honest significant difference (HSD) adjustment, applied to control for multiple comparisons. Finally, mixed effects ordinal logistic regression analyses were used to ascertain the likelihood of participants reporting different stimulation factors (e.g., “how long did stimulation last?”) within each group. All models were checked for standard diagnostics such as normality of the residuals and the random effects. Due to its non-normality, the total errors variable was analysed using the natural logarithm of total errors plus 1, where the addition of 1 to all total errors is used to avoid numerical errors among those with 0 total errors. A 5% significance level and two-sided tests were used throughout.

## Results

### Memory performance

**Figure 3:**
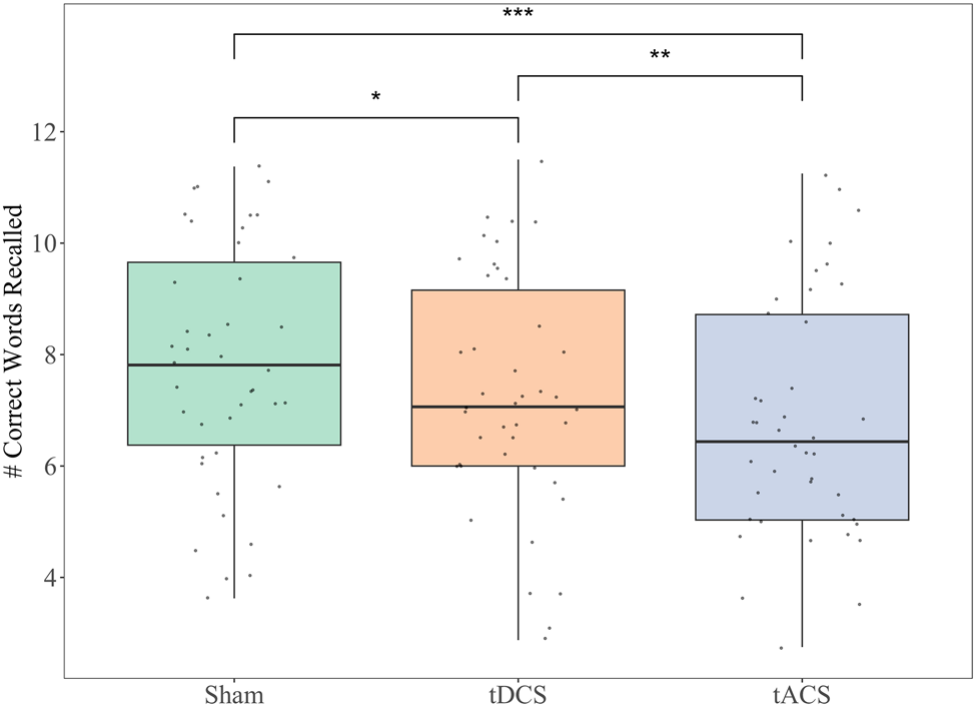
Average number of correct words recalled during each stimulation type with vertical lines representing standard error. *p ≤ 0.05; **p ≤ 0.01; ***p ≤ 0.001.

Figure 3 shows the average words recalled during each stimulation type. A significant effect of stimulation type on memory performance was found [*F*(2,964) = 26.27, *p* < 0.001]. Post hoc analyses revealed that fewer words were recalled during tDCS (*M* = 7.3, *SE* = 0.32; *p* < 0.001, 95% CI for the difference = [− 0.85, − 0.21]) and theta tACS (*M* = 6.8, *SE* = 0.32; *p* < 0.001, 95% CI for the difference = [− 1.33, − 0.68]) when compared to sham stimulation (*M* = 7.8, *SE* = 0.32), respectively. Participants also recalled significantly fewer words during tACS compared with tDCS (*p* = 0.002, 95% CI = [− 0.80, − 0.15]).

### Intrusion and repetition errors

**Figure 4:**
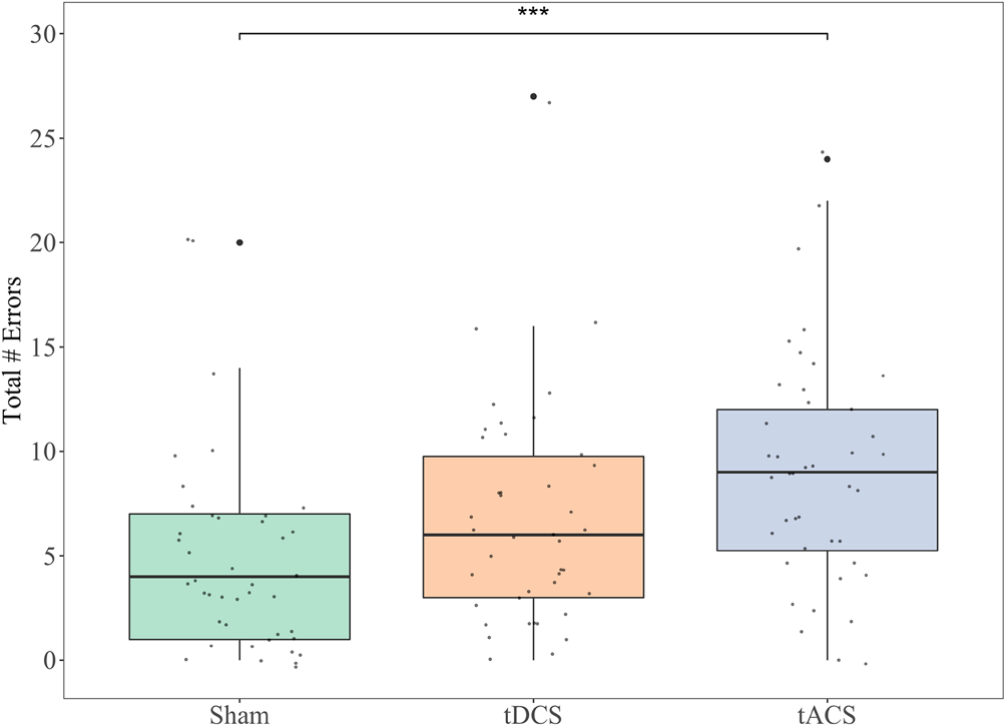
Average total number of errors (intrusion and repetition) during each stimulation type with vertical lines representing standard error. *p ≤ 0.05; **p ≤ 0.01; ***p ≤ 0.001.

Figure 4 shows the average number of errors during each stimulation type. A significant main effect of stimulation type on the total number of intrusion and repetition errors was found [*F*(2,82) = 10.19, *p* < 0.001], with post hoc analyses revealing that more errors were made during theta tACS (*M* = 9.1, *SD* = 5.5) compared with sham stimulation (*M* = 4.8, *SD* = 4.7; *p* < 0.001, 95% CI = [0.27, 0.87]). The comparison between tACS and tDCS (*M* = 6.8, *SD* = 5.3) approached significance (*p* = 0.064, 95% CI = [− 0.01, 0.59]), with more errors committed in the former group. There was no statistically significant difference between the tDCS and sham group, although this also trended towards significance (*p* = 0.071, 95% CI = [− 0.02, 0.58]).

To further investigate the impact of stimulation on memory errors, we analysed both intrusion and repetition errors separately. We found a significant main effect of stimulation type on intrusions [*F*(2,82) = 5.81, *p* = 0.004], with post hoc analyses revealing significantly more errors during theta tACS (*M* = 3.4, *SD* = 2.3) compared to sham stimulation (*M* = 1.8, *SD* = 2.2; *p* = 0.003, 95% CI = [0.48, 2.71]). There was also a significant main effect of stimulation type on repetitions [*F*(2,82) = 8.70, *p* < 0.001], with significantly more errors occurring during tACS (*M* = 2.3, *SD* = 1.7) compared to both tDCS (*M* = 1.5, *SD* = 1.7; *p* = 0.017, 95% CI = [0.11, 1.45]) and sham stimulation (*M* = 1.2, *SD* = 1.3; *p* < 0.001, 95% CI = [0.47, 1.81]). There we no other significant differences between groups (all *p*-values > 0.414).

### Impedance, perceived intensity and side-effects

There was no statistically significant main effect of stimulation type on impedance [*F*(2,82) = 2.19, *p* = 0.119]. Perceived stimulation intensity was statistically significantly different between stimulation groups [*F*(2,82) = 3.81, *p* = 0.026]). Post hoc analyses revealed a statistically significant difference between tDCS and sham stimulation, with perceived intensity higher in tDCS (*p* = 0.024, 95% CI = [0.11, 1.84]). There were no statistically significant differences when comparing other groups, including theta tACS vs sham (all *p*-values > 0.145).

In terms of overall side-effects of stimulation, there was a statistically significant main effect of stimulation type [*F*(2,82) = 8.72, *p* < 0.001]. Post hoc analyses revealed that participants perceived significantly stronger side effects during tDCS compared to both tACS (*p* = 0.004, 95% CI = [− 0.38, − 0.06]) and sham stimulation (*p* = 0.001, 95% CI = [0.10, 0.41]). There was no statistically significant difference between theta tACS and sham stimulation (*p* = 0.876, 95% CI = [− 0.13, 0.19]).

Mixed effects ordinal logistic regression analyses found that participants were 16.2 and 8.1 times more likely to perceive stimulation as longer during tDCS and theta tACS compared to sham stimulation, respectively (*p* < 0.001, 95% CI = [4.94–53.16]; *p* < 0.001, 95% CI = [2.80–23.53]). Participants were also 3.8 times more likely to report that stimulation impacted their performance during tDCS compared to sham stimulation (*p* = 0.014, 95% CI = [1.31–10.78]). No significant differences were found between any of the other groups on these measures, including tACS vs sham (all *p*-values > 0.147).

## Discussion

The present study investigated whether anodal tDCS and theta tACS over the left temporal lobe improved episodic memory performance in healthy adults. Unlike many previous studies comparing the effects of tDCS and tACS on memory, we applied stimulation to the temporal lobe, as encoding and recall of information is typically associated with temporal lobe structures (e.g., the hippocampus and entorhinal cortex). We anticipated that both stimulation types would result in improved memory performance compared to sham stimulation, with theta tACS resulting in superior performance compared to tDCS.

In terms of memory performance, our findings did not support our initial hypotheses. Instead, they demonstrated the opposite effect, with participants recalling significantly fewer words during tDCS and tACS, compared to sham stimulation. Furthermore, participants performed significantly worse during tACS compared to tDCS, a finding that was unexpected given the breadth of literature demonstrating the enhancing effect of tACS on memory performance^2,10,24^. While we theorised that theta tACS would enhance memory performance, as theta activity is known to underlie successful encoding and recall of information (i.e., theta band activity)^39–41^, our findings suggest that applying theta activity to the left temporal lobe has a strong deleterious effect on memory, beyond that of tDCS. One explanation for our findings is that stimulation, particularly theta tACS, disrupted well-functioning memory circuitry in our young, neurologically healthy sample. A recent study by Klink et al.^42^ investigated the effects of tDCS and theta tACS on associative memory in older adults and found that the beneficial effects of tACS were only evident in older participants within their sample, with no effect in younger older adults or when younger and older groups were combined. They suggested that the specific beneficial effect of theta stimulation in older adults may be due to the chronically reduced synchrony or power of theta networks that is associated with aging^43–45^. This may explain our findings, as stimulation in our sample, which was even younger than that of Klink et al.^42^, had a deleterious effect, rather than no effect—possibly indicating that theta stimulation disrupted memory processes that we would expect to be functioning normally in younger adults^10^.

Participants made significantly more memory errors (i.e., total intrusions and repetitions) during theta tACS compared to sham stimulation, with the comparison to tDCS trending towards significance. After separating intrusion and repetition errors, we found that tACS resulted in more intrusion errors, compared with sham stimulation, and more repetition errors compared to both sham and tDCS. While both intrusion and repetition errors are typically associated with fronto-subcortical patterns of impairment^46,47^, a number of studies have demonstrated increased errors in the context of MTL dysfunction^48–50^. A recent study by Graves et al.^51^ however demonstrated that these error types were associated with dysfunction in distinct regions when investigated separately—with intrusion errors associated with damage to the MTL, and repetition, or perseverative errors, associated with frontal impairment. In the context of memory improvements, the DLPFC has been targeted as it is functionally connected to the MTL^52,53^, and thought to be more easily accessible with non-invasive stimulation, compared to other mesial structures (such as the hippocampus and entorhinal cortex). The specific impact of tACS on repetition errors, compared to both sham and tDCS, may represent the disrupted communication between the MTL and PFC—something that is thought to occur through specific neural oscillations, such as theta activity^18,19^. Alternatively, stimulation may have disrupted memory processes by directly engaging MTL structures (as depicted by our modelling prediction; see Fig. 2). Theta tACS may have been more disruptive, compared with tDCS, due to the interference of theta oscillatory activity known to underlie successful memory processes. Unfortunately, we can only speculate on this frequency-specific explanation, as we did not explore the impact of tACS at a different frequency that is not typically associated with cognition.

There was no significant difference in stimulation impedance between groups, suggesting comparable conductivity levels between stimulation types. Perceived stimulation intensity and overall side-effects of stimulation were however higher in the tDCS group compared to both tACS and sham stimulation. While the perceived intensity and side-effects of stimulation may have impacted performance, it is worth noting that tACS (which did not significantly differ from sham stimulation on these factors) was associated with a greater impact on memory performance. The effectiveness of the neuroConn sham protocol has previously been investigated and was found to be an adequate blinding method, whereby 73% of the sham group in a double-blinded study believed they had received active tDCS^54^. Furthermore, recent research has indicated that correct sham guessing does not moderate the effect of tDCS on post-stimulation memory performance^55^.

Therapeutic transcranial electrical stimulation is of growing interest, particularly in the context of memory-related disorders (e.g., AD and TLE). However, there are several different stimulation modalities available. Additional research is required to understand which stimulation modalities produce reliable improvements in cognition. Our findings highlight how different stimulation methods can differentially impact cognition, as theta tACS was associated with worse memory performance, compared to tDCS. Furthermore, the target of stimulation often varies between studies, with the majority of tDCS research focussing on the stimulation of the DLPFC. Our study applied current to the temporal lobe to engage mesial temporal structures that are typically associated with encoding and recall of information. Our findings appear to support the role of these structures within memory processes; however, contrary to our hypotheses, we did not demonstrate an enhancing effect—instead reducing memory performance during stimulation. These finding have major implications for how we utilise therapeutic stimulation in future studies, as they indicate that some stimulation modalities, namely tACS, may only be beneficial in certain cohorts (i.e., older adults) or under certain conditions (i.e., where specific brain activity is expected to be aberrant or diminished).

There were a few limitations to our study. First, given our study did not include individual MRIs, it is difficult to confidently ascertain whether the current flow of stimulation engaged our region of interest. Nevertheless, the use of an MRI-derived standard head model (i.e., New York Head) is a well validated method for predicting current flow in the absence of individual MRI scans^35,36^. Second, participants reported significantly higher stimulation intensity during tDCS compared to sham stimulation, suggesting they were able to tell the difference between these stimulation conditions. While this may have impacted performance during tDCS, it does not explain the greater impact of tACS on memory performance. Finally, while our findings suggest that theta tACS has a larger deleterious effect on cognitive performance than tDCS, it is difficult to infer how much of this effect is due to the use of the theta frequency without a comparative tACS control condition. Future studies should aim to investigate the impact of frequency by comparing theta stimulation to a control frequency that is not typically associated with cognition, as well as active tDCS.

## Conclusions

In conclusion, our findings indicate that TES over the temporal lobe can have a deleterious effect on memory processes (word recall and errors). Furthermore, we demonstrated that theta tACS was associated with more significant impairment to memory processes compared to tDCS and sham stimulation. This has important implications when further exploring the therapeutic effects of TES on memory performance. Future research should attempt to replicate these effects with different tACS frequency conditions, to determine the specific impact of theta frequency on memory performance.

## Data Availability

The datasets generated during and analysed during the current study are available from the corresponding author on reasonable request.
